# Withdrawal Aversion and the Equivalence Test

**DOI:** 10.1080/15265161.2019.1574465

**Published:** 2019-03-21

**Authors:** Dominic Wilkinson, Ella Butcherine, Julian Savulescu

**Affiliations:** 1 University of Oxford,; 2 John Radcliffe Hospital,; 3 and Murdoch Children’s Research Institute; 4 University of Melbourne

**Keywords:** Critical Care/Ethics,, Ethical Analysis,, Medical Ethics Medical,, Passive Euthanasia,, Withholding treatment/Ethics

## Abstract

If a doctor is trying to decide whether or not to provide a medical treatment, does it matter ethically whether that treatment has already been started? Health professionals sometimes find it harder to stop a treatment (withdraw) than to refrain from starting the treatment (withhold). But does that feeling correspond to an ethical difference? In this article, we defend equivalence—the view that withholding and withdrawal of treatment are ethically equivalent when all other factors are equal. We argue that preference for withholding over withdrawal could represent a form of cognitive bias—withdrawal aversion. Nevertheless, we consider whether there could be circumstances in which there is a moral difference. We identify four examples of conditional nonequivalence. Finally, we reflect on the moral significance of diverging intuitions and the implications for policy. We propose a set of practical strategies for helping to reduce bias in end-of-life decision making, including the equivalence test.

## DOES EQUIVALENCE MATTER?

In a previous article, we defended the view that withholding (WH) and withdrawal (WD) are ethically equivalent (Wilkinson and Savulescu 2012), a view denoted by Sulmasy as “The Equivalence Thesis” (Sulmasy and Sugarman 1994). However, not all ethicists or health professionals agree with that conclusion. Lars Ursin, writing in this issue, rejects the equivalence thesis, at least in some situations (Ursin 2019). He claims that the equivalence thesis shows a lack of respect for the intuitions of practising health professionals and lacks understanding of the nuance and context of medical decision making, and that factors such as patient autonomy and physician responsibility mark a moral distinction between stopping and not starting treatment.

Ursin does not make clear what the implications would be of his own view about the nonequivalence of WH and WD. It may be helpful to consider some cases (Box 1).^1^

**Box 1. Case examples: Withholding and withdrawing**

**Case 1**. Nontreatment in best interests

An extremely preterm infant, Paula, is delivered at 23 weeks gestation following rapid preterm labor. Prior to delivery doctors had estimated that she had approximately a 40% chance of survival (and if she survived, a 15–20% chance of severe neurological impairment). Doctors had attempted to counsel Paula’s parents prior to delivery. They would have been prepared to withhold resuscitation if her parents had wished this. However, because of the rapidity of the delivery, Paula’s parents had little time to consider their situation; they asked doctors to do all possible to save the infant. Paula is resuscitated and taken to intensive care. She is unwell in the first several days of life. At a week of age, Paula’s parents take the professionals aside. They have been thinking hard about the situation, have spoken to their wider family, and no longer feel that it is the right thing to do to keep her alive with intensive care. On reflection, they feel that if they had had enough time to consider their decision, they would not have desired resuscitation at birth. The health professionals consider Paula’s clinical condition. They estimate that Paula currently has a 40% chance of survival and (if she survives) has a 15–20% chance of severe neurological impairment. The health professionals explain to the family that although they would have been prepared to WH intensive care at delivery given this prognosis, they are now not willing to WD intensive care unless the infant has a much higher chance of death, or a much higher chance of severe impairment.

**Case 2** Nontreatment because of limited resources

A 6-month-old infant, Theo, has a congenital genetic disorder. Doctors are considering whether he should be eligible for a new but highly expensive medicine that is in very short supply within the public health system but might prolong Theo’s life. Theo’s parents are very keen for him to receive the treatment. Given the cost of treatment, a policy has been developed that would provide it for a limited number of infants with certain forms of the disorder who do not have other serious comorbidities. (In such cases, patients would need to pay for treatment privately or travel overseas.) Theo meets the eligibility criteria for access to the medication, but a month after he is commenced on treatment, he has a respiratory arrest and sustains significant hypoxic ischemic brain injury. He no longer meets the eligibility criteria for funding of the treatment. If he had suffered the hypoxic brain injury two months earlier, treatment would not have started. However, Theo’s parents remain keen for him to continue the treatment, and his doctors are reluctant to stop the treatment since it has already commenced.^2^

What would it mean to say that WH and WD are “ethically equivalent”? As we argued previously, (and as accepted by Ursin), the equivalence thesis is best understood as a statement about comparative permissibility: If it is permissible to WH treatment it would also be permissible to WD the same treatment (if already started, and all other things being equal). On this view, it would appear to be ethically acceptable for the doctors in Case 1 and Case 2 to stop treatment.

**Figure 1:**
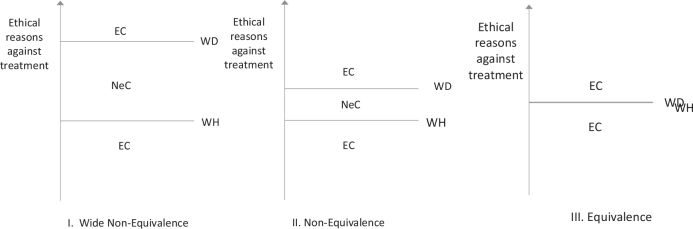
Ethical reasons and equivalence cases. According to nonequivalence (View II), there need to be stronger ethical reasons to withdraw (WD) treatment than to withhold (WH). There could be cases where there is sufficient reason to WH, but not to WD (nonequivalence cases, NeC). The number of NeC depends on how large a difference is observed between WH and WD. On wide nonequivalence views (I), there would be many NeC since it is much harder to justify withdrawing than withholding. There could be equivalence cases (EC) where both WH and WD would be permissible or impermissible on any of these views.

By contrast, nonequivalence endorses the idea that there could be situations where it would be permissible to do one but not the other (call these nonequivalence cases). The most common nonequivalence view holds that WD is a more morally serious decision than WH treatment. There would need to be more moral reason to stop treatment than not start it. The doctors in the hypothetical cases in Box 1 appear to believe that those situations are nonequivalence cases. Figure 1 illustrates these views graphically.

The debate about equivalence and nonequivalence matters because there is a disconnect between ethical theory and the views of health professionals. Many doctors and nurses observe an ethical difference between WH and WD, while most professional guidelines endorse the equivalence thesis (Sprung et al. 2014; Wilkinson and Savulescu 2012). If nonequivalence is incorrect, it may represent a form of cognitive bias. We could call this withdrawal aversion:

*Withdrawal aversion*—a nonrational preference for withholding treatment over withdrawal of treatment.^3^

Withdrawal aversion could have several serious ethical consequences. First, it may mean that patients have harmful or nonbeneficial treatment prolonged when it should have been stopped. That may also have the effect (as we argued in our previous article; Wilkinson and Savulescu 2012) that limited health care resources are distributed unjustly, and other patients are denied treatment. Second, it may mean that some patients have treatment unjustly withheld when it should be provided. Because doctors know (or think) that once they have started treatment it will be very difficult to stop, they are unduly restrictive in providing treatment. For example, they may decide not to admit a patient to intensive care, decide to withhold resuscitation, or decide not to start an expensive treatment even though in fact that treatment would be desired and potentially in the patient’s interests.^4^

How big a problem withdrawal aversion poses may depend on how large an ethical difference is observed between WH and WD. Some professionals may feel that there is a small ethical difference between WH and WD. On such a view, nonequivalence cases would occur infrequently. Other professionals may observe a large ethical difference (wide nonequivalence, Figure 1): Much greater ethical reason is required to WD treatment, so they permit WD of treatment only rarely.^5^

On all of the views that we have described, there are some situations where there is a sufficiently strong ethical reason not to treat that either WH or WD would be permissible.^6^

## IN DEFENSE OF EQUIVALENCE

Where treatment is not being provided because the patient does not desire it, or because that treatment is not in the patient’s best interests, it is hard to see how the mere fact that the treatment has already been started could make an ethical difference.

In a situation like Case 1 (Box 1), the salient ethical features appear to be same. Whether treatment is withheld or withdrawn, the outcome of nontreatment is the same (likely death) and the intention of the doctors is the same (to act in Paula’s best interests and respect her parents’ wishes). How could it make a difference to her best interests?

Some may feel that, given her prognosis, life support would be in Paula’s best interests—in that case it would be wrong for the doctors to allow her to die either by not resuscitating, or by withdrawing treatment. Others may feel that life support is not in Paula’s best interests, given her prognosis and her parents’ wishes (Wilkinson 2010)—in that case it would be ethical to allow her to die either by withholding or by withdrawing treatment.

What is more, the cause of Paula’s death is the same (extreme prematurity and respiratory insufficiency), and the responsibility of her doctors for the decision is the same (they are responsible for making an ethically justified decision in her best interests).

As noted in the preceding, withdrawal aversion in a case like Case 1 could have harmful consequences. It may also have absurd consequences. It may mean, for example, that if Paula’s ventilator became accidentally disconnected, her doctors feel justified in not reconnecting it (though they would not withdraw treatment). Or it may mean that in the delivery room, had her parents changed their minds (and asked for their baby not to be resuscitated) before insertion of a breathing tube, this would have been respected, but if they had made the same request only a couple of minutes later, it would be ignored. That, to many people, including ourselves, seems absurd.

Some people who support nonequivalence (and perhaps Ursin shares this view) may feel that in a case of nontreatment in the patient’s best interests (or at patient request), WH and WD would be ethically equivalent. This does not disprove nonequivalence (NE)—since there can still be equivalence cases on such a view (Figure 1). However, they may feel that cases like Case 2, involving nontreatment for reasons of limited resources, are nonequivalent.

In our previous article, we considered and rejected a series of possible reasons for thinking that resource allocation would justify nonequivalence.(Wilkinson and Savulescu 2012) Here, we consider three reasons that Ursin believes support his view.

### Acts and omissions

Ursin argues that “there is a moral difference between letting something happen versus actively causing the same thing”; he appears to regard WD as an action and WH as an omission and sees this as a reason to treat them differently. However, this is by no means obvious. While the acts/omissions distinction is related to debates about WH and WD, it is conceptually separate. Many ethicists, and most legal jurisdictions, regard both withdrawal of treatment and withholding of treatment as omissions (McGee 2005).^7^ It is for that reason that many jurisdictions will permit doctors to withdraw treatment though they will not permit doctors to actively end the lives of their patients (even when that would be in the patient’s best interests and respect their wishes) with a lethal injection. If Ursin were correct that withdrawal of treatment were an “action,” that should mean that such jurisdictions are either wrong to allow treatment withdrawal, or wrong to prohibit active euthanasia.

More importantly, even if it were true that stopping treatment is an instance of more direct causation than not starting treatment,^8^ there would have to be a further reason why this matters in cases of nontreatment because of limited resources. Either it is justified to decide to allocate treatment to Theo or it is not justified. How does it make any difference whether he has already started on the drug?

### Autonomy

Ursin claims that patient autonomy is more compromised by WD treatment than by WH treatment because “the patient has attached further activities and life paths” to existing options and treatment, and consequently WD represents a greater restriction of the patient’s liberty. However, this seems to be a weak argument in favor of nonequivalence. For one thing, if WD of treatment were associated in some cases with greater goal frustration, that would mean that “other things were not equal” (i.e., it would not undermine equivalence). It would also be unlikely to apply in the types of cases where this issue is most relevant—stopping or not starting life support in intensive care. For an unconscious adult patient attached to a ventilator, or for a very young child (as in the cases in Box 1), there are no patient preferences or plans that arise as a consequence of starting treatment.^9^ Moreover, the very nature of rationing of medical treatment is that this constitutes an infringement of patients’ liberty and autonomy. If it is justified to allocate scarce medical treatment in a way that some patients are denied treatment, that will inevitably mean that some patients’ desires and plans are frustrated, so their liberty is compromised. Patients do not have a positive right to demand medical treatment from a public funded health care system—their needs and desires and liberty compete with the needs, desires, and liberty of other patients.

### Responsibility

Ursin claims that in commencing a treatment physicians have taken on a responsibility toward a patient. Stopping that treatment would involve “relinquishing” responsibility—something that he contends “is not straightforward” (Ursin 2019). Such a claim might make sense in some instances of WH and WD treatment. Some may feel, for example, that a physician who receives a telephone referral from a different hospital about a seriously ill patient has no specific responsibility to the care of that patient. However, if the physician accepts a patient into his or her intensive care unit, this takes on a fiduciary responsibility toward the patient. In our previous article, we consider this argument and suggest that those who provide this sort of medical treatment have duties to patients both within and outside the intensive care unit. However, even if that isn’t accepted, Ursin’s responsibility argument would appear to have no application to cases like Case 2. In that example, the doctors providing the treatment to Theo had an existing therapeutic relationship with him and his family before commencing the expensive treatment. That relationship would continue (it would not be relinquished) if the treatment were stopped. Theo’s doctors’ responsibility toward him might make it difficult for them to stop treatment after his condition changed (and he was no longer eligible), but it would also have been difficult for them to withhold treatment before it had started. Furthermore, such questions of responsibility might be easily sidestepped by making decisions about treatment at a higher level. For instance, the decision to fund treatment for Theo could be made be a specialized treatment panel (Wilkinson and Savulescu 2018). It isn’t clear that such a panel would have acquired a responsibility by commencing treatment. Furthermore, they might well have communicated to his family that ongoing provision of treatment was dependent on him continuing to fulfill eligibility criteria.

## CONDITIONAL NONEQUIVALENCE

While we have defended the view that there is no intrinsic ethical difference between WH and WD treatment, we have some sympathy with Ursin’s view there are some special situations where health professionals would be justified in treating WH and WD decisions differently. These special exceptions do not invalidate the equivalence thesis, since one important part of that claim is the ceteris paribus clause: WD and WH are equivalent *if all other things are equal*. Here we identify and describe situations where other things are not equal.

### Prognostic nonequivalence

As we noted in our original article, and as pointed out by Ursin, the outlook for patients who have already commenced on treatment is not necessarily identical to the outlook of those who have not yet started. Where a patient’s prognosis would be different, WH and WD would not be equivalent. For example, if the estimated chance of survival for infant Paula in Case 1 were significantly better at a week of age, that would provide a good reason not to WD treatment now.^10^ Prognostic nonequivalence would not in general favor withholding treatment over withdrawal of treatment—in many circumstances the opposite would be true. Access to additional prognostic information (including response to treatment) would potentially favor WD over WH since the outcome would be less uncertain.

### Preference nonequivalence

One of the ethical reasons not to provide treatment can be that the patient (or surrogate on the patient’s behalf) expresses that he or she does not want treatment. In some of these cases, the patient or family may not regard WH and WD as equivalent.^11^ The family or the patient may be prepared to WH treatment, but not agree to WD. That would provide an ethical reason to favor WH over WD in those situations.

It is worth pointing out that this preference does not amount to an intrinsic ethical difference between WH and WD.^12^ It also would not rule out WD in other situations where patient or family preferences are not decisive. For example, there is widespread ethical acceptance of the idea that parents’ wishes about medical treatment for their child should often be respected, but they should not be able to demand treatment that would cross a certain “harm threshold,” that is, would pose a significant risk of serious harm to a child (Diekema 2004). In such situations, parents’ desire for the treatment is not ethically relevant—doctors should not provide that treatment no matter how much parents want it. However, if that is the case, it would be a mistake to adopt a different harm threshold for withdrawing harmful treatment from one applied to withholding harmful treatment. The fact that a treatment has already started would not make it any less harmful to the child.^13^

Family or patient preferences for withholding over withdrawal would also not be decisive if the reason to not provide treatment is because of limited resources. When health systems are deciding whether to provide treatment or who to provide treatment to, they might choose to allocate to those in greatest need of treatment, or to those who will benefit the most from treatment. However, it would appear to be a mistake (both epistemically and ethically) to try to allocate based on who has the greatest desire for treatment.^14^

### Ownership nonequivalence

In some cases, provision of a treatment to a patient might appear to confer ownership. If a public health care system withdrew an artificial leg or set of spectacles from a patient, it does seem likely that the patient would feel much more aggrieved than if that leg or those spectacles had never been provided. This sort of situation might arise in particular with prosthetics or other devices (e.g., spectacles, wheelchairs) that have been custom made or modified for the patient, and that the patient has come to understand or expect will remain available for his or her use. It would not apply to many medical treatments—those that require continual supply, or that are usually shared between many patients. For example, receiving a drug prescription cannot in any obvious way generate an ownership claim over future supplies of the drug. Likewise, there is no sense in which a patient attached to a ventilator in intensive care “owns” that machine. Furthermore, in many situations, health systems may explicitly provide medical equipment as a loan. In those cases, while the patient might still feel that he or she has some special claim to the equipment, it is hard to see that the patient is justified in upholding that claim. Historical entitlement theory does not give rise to moral claims in situations where the allocation of resources is unjust (Harris 1994).

### Bodily integrity nonequivalence

Finally, there are a small number of medical treatments where the physical process of withdrawing the treatment would involve an invasive or intrusive procedure. Take, for example, an implanted pacemaker or Berlin heart. If a patient has had such a device implanted or attached and wishes it to continue, but the physician decided to withdraw it, the doctor would have to restrain the patient, sedate or anesthetize the patient against his or her will, and then remove the device. Even staunch defenders of equivalence would accept that this looks ethically much more significant than a physician or surgeon simply refusing to implant such a device. Again, this doesn’t invalidate equivalence. The psychological and physical effects on the patient of holding the person down to remove the device would mean that other things are not equal.

One interesting question is whether withdrawal of an endotracheal tube constitutes an infringement of bodily integrity (such that WD is worse than WH). At least for a patient who is sedated and unconscious in intensive care, neither removal of a breathing tube nor disconnection of a ventilator is an intrusive or invasive procedure (paradoxically, removing them they might be seen to restore bodily integrity). In Case 2, if the treatment for Theo had been a ventilator rather than a life-prolonging medication, it is hard to see how stopping ventilation would affect his bodily integrity any more than stopping a medication.

## INTUITIONS, REFLECTIVE EQUILIBRIUM, GUIDANCE

We contend that ethical arguments strongly favor the equivalence thesis. In most situations, decisions to stop treatment and decisions not to start treatment are ethically equivalent. However, surveys of health professionals indicate that they are not convinced—they have a strong intuitive sense that withdrawal of treatment is more serious than withholding (Chung et al. 2016; Wilkinson and Savulescu 2012). What should we do with the mismatch between ethical theory and intuitions?

To a large degree, that depends on your view on ethical analysis. According to ethical intuitionists, “gut feelings” can provide evidence of fundamental moral truths (McMahan 2013). Intuitions have epistemic moral authority, and therefore these intuitions of health professionals that WD and WH are ethically distinct indicate that the equivalence thesis is wrong. However, intuitions, including those of health professionals, can be misguided—influenced by factors that are not relevant, or by prejudice. They may change over time. In a large survey of the British public in 1983, 50% indicated a belief that sexual relations between two adults of the same sex was always wrong (NatCen Social Research 2013). Presumably (though we are not aware of specific evidence to corroborate this), many health professionals shared this view. Three decades later, the proportion of the population endorsing that had fallen to 22% (NatCen Social Research 2013).

The opposite (theoretical) approach to ethics derives moral norms through identifying the correct moral theory and applying that to a particular situation. A theoretical approach to ethical enquiry would suggest that intuitions have no epistemic force; if intuitions diverge from the results of ethical analysis, they are simply mistaken. On such a view, health professionals are wrong to hold on to nonequivalence, and should abandon their intuitive allegiance to the distinction between withholding and withdrawing. However, it is challenging to identify the correct moral theory. We need some way of assessing different contenders.

Our own preference, as with many other contemporary ethicists, is to dry to draw on both ethical theory and ethical intuitions in a process that John Rawls referred to as “reflective equilibrium” (Rawls 1999). This uses ethical intuitions to help reflect on and derive ethical theory to apply to problems. It also involves critically evaluating our own intuitions in situations where they appear to diverge from the results of theoretically informed ethical analysis.

The methodology of reflective equilibrium does not always make it clear what to do when theory and intuitions clash. Should we revise the theory or revise the intuitions? Our own view is that we should do the latter in the case of withholding and withdrawing medical treatment. That is because we think that the arguments in favor of equivalence are strong, clear, and hard to reject. Moreover, drawing a distinction between WH and WD has harmful consequences for the patient and for distributive justice. It means some patients are not given a trial of treatment that might benefit them, while other patients are kept on treatment that is not in their interests or is precluded by distributive justice. As we discussed in our previous article, there are also other confounding psychological explanations of the widespread nonequivalence intuition. In particular, status quo bias and loss aversion are common and have a substantial (though often irrational) effect on decision making. Withdrawal aversion may be a manifestation of these cognitive phenomena. However, we accept that others will disagree. Their evaluations of the ethical arguments may differ from our own. They may not share our own sense that it would be incoherent, inconsistent, and potentially harmful to apply a higher threshold to decisions to withdraw than to decisions to withhold treatment.

That, then, raises a further question. What should policymakers do in the face of ethical disagreement? One important step is to acknowledge ethical complexity. Ursin is correct to note that existing policies and guidance wrongly imply that all ethicists support equivalence. Clearly, they do not. In the face of ethical dissensus (Wilkinson and Savulescu 2018), one plausible ethically pluralist approach would be to allow individuals to decide for themselves how to weigh up the ethical considerations and how to apply them to their own lives. That would mean that patients’ or surrogates’ views about withholding and withdrawing should be respected—at least (as noted in the third section of this article) in situations where their views and interests are the primary ethical consideration. However, that does not mean that health professionals should be free to apply their evaluation of equivalence/nonequivalence to decisions. It would be wrong for doctors to apply a higher threshold to treatment withdrawal if the patient does not share the doctor’s preference for treatment withholding.

It also does not follow that policies or guidelines should remain completely agnostic in the face of disagreement. There are many ethical issues on which there can be different ethical viewpoints. It is entirely within the realm of those writing professional guidelines to evaluate those different viewpoints and to come down on one side or the other. There is a need to guide professionals in situations where the patient’s views about withholding/withdrawing are not clear, or, as noted earlier, where the patient’s/family’s views are not decisive. Guidelines might provide practical strategies for professionals in identifying withdrawal aversion and addressing it (Box 2).^15^

In particular, there is a need to decide collectively how to manage limited resources (Wilkinson and Savulescu 2018). While it may be psychologically more difficult to stop treatment than to not start it, it is a potentially costly mistake to separate withholding and withdrawing decisions. There is a critical need to evaluate carefully how to fairly allocate resources. But such allocation should not give preference to those currently receiving treatment. The critical ethical issue, the one that needs most attention, is whether or not treatment should be provided—not whether treatment has previously been started.

**Box 2. Practical strategies for overcoming withdrawal aversion**

1. The equivalence test: If patient P1 is currently receiving treatment—imagine that another patient P2 were to present tomorrow with identical features to P1 (identical preferences, illness, prognosis etc). Would you be prepared to withhold treatment from P2? If so, on the basis of ethical consistency you should be prepared to withdraw treatment today from P1.
2. The “if I’d only known” test. If patient P is currently receiving treatment, think back to before this started. Imagine that you knew then what you know now about the patient (in terms of response to treatment, prognosis, etc.), would you have been prepared to withhold treatment then? If so, you should be prepared to withdraw treatment from P now.
3. The escalation reversal test. In situations where a treatment can be provided at different levels of intensity, patient P is currently receiving treatment at level T, and you are not prepared to withdraw or reduce T. (For example, the patient is receiving a certain number or level of organ support.) Are you prepared to withhold further escalations of treatment to T+ (e.g., not institute additional organ support, not add additional inotrope)? If prepared to withhold T+, imagine that P were already at T+. Would you be prepared to reduce the patient’s support down to level T? If you would not be prepared to reduce T + to T, that may imply a bias for the status quo (Bostrom and Ord 2006).
4. The peer review test. In a situation where you are not prepared to withdraw treatment (but would withhold), consider whether any of your professional peers (e.g., other specialists) would withdraw treatment in a patient with features similar to the current patient. If so, you should consider whether your own personal values are influencing their ethical evaluation. You should potentially offer withdrawal of treatment as an option, or referral to another physician (Wilkinson and Truog 2013).
5. Conditional offer of treatment. At the time of commencing therapy, identify the goals of treatment, along with potential reasons (triggers) to discontinue treatment. Offer treatment on the condition that measurable progress toward those goals is able to be discerned within a set period, and that discontinuation triggers have not been observed. After the set period, if the conditions are not met, withdraw treatment.^16^
6. Defined treatment period. Provide treatment for a set period. At the end of that period, the default would be for treatment to be withdrawn. There would need to be an active decision to reinstitute therapy, or to embark on a further period of treatment.

## Footnotes

1. These cases both relate to the care of newborn infants. That reflects the clinical experience of one of the authors (DW). (The cases are fictional but based on composite real cases.) It also provides a counterpoint to Ursin’s cited empirical research in newborn intensive care (Ursin 2019). However, as noted in our previous article, equivalence is equally relevant in the medical care of adults.

2. In this article, we are not defending the policy about allocation of treatment. Some may view it as discriminatory to deny patients treatment on the basis of their comorbidities. However, on such a view, it would be discriminatory either to WH or to WD treatment.

3. This cognitive bias is potentially akin to the psychological phenomenon of loss aversion, wherein “losses loom larger than improvements or gains” (Kahneman et al. 1991).

4. Withdrawal aversion might be one reason for trials of experimental treatment not being provided in some high-profile cases of conflict over treatment (Wilkinson and Savulescu 2018).

5. Since Ursin does not provide any examples of cases in which he endorses nonequivalence, it is difficult to know whether he endorses wide nonequivalence or a narrower form

6. This does not apply to the most extreme form of a wide nonequivalence view, which holds that while WH of treatment is sometimes permissible, WD of treatment is never permissible. There are some religions that appear to endorse this view—for example, orthodox Judaism (Kunin 2010).

7. For example, Jeff McMahan and Frances Kamm have argued that withdrawing lifesaving aid is a form of “letting die” rather than killing (Kamm 2001; McMahan Airedale NHS Trust v BlandAiredale NHS Trust v Bland^1993^). In the highly influential UK court case of Tony Bland, the law Lords concluded that “the doctor's conduct in discontinuing life support can properly be categorised as an omission” (1993). Others may regard WH and WD both as “actions,” given that the doctor makes a deliberate decision that predictably and intentionally changes the outcome.

8. One of us has argued that withdrawal of life-sustaining treatment is a form of killing, though that does not make it impermissible (Savulescu 2013).

9. Ursin might point to the preferences and plans of family members in such cases—grounding a parental or family autonomy argument against WD. However, that would imply that the reason not to WD treatment is contingent on the strength of preferences of the family. There might be cases where some families (whose child has not yet started treatment) have desires for the treatment, or have already made plans based on that treatment, that are much stronger or more well-formed than other families for whom treatment has not yet commenced. (It would perhaps incentivize families to make advance plans for treatment in order to strengthen their case to be provided with it.) In such cases, the autonomy argument would favor WD of treatment from the existing patients.

10. In practice, it can be difficult to know for an individual patient what the prognosis is, both before and after starting treatment. This presents an epistemic challenge for implementing the equivalence thesis. In some places in his article, Ursin appears to make the stronger claim that provision of treatment will always change a patient’s prognosis—so that in fact the ceteris paribus clause never holds. We would suggest that it is not practically or ethically necessary to know that a patient’s prognosis currently is identical to that before treatment. It is enough to know that the patient’s outcome is at an approximate level that would have justified withholding.

11. Families may often manifest withdrawal aversion. Although we are not aware of any surveys of the general public, in our personal experience of families in intensive care (DW) many such families find decisions to WH treatment easier than decisions to WD.

12. Patients might have a preference for a liquid form of a medicine over tablets. Doctors should often respect that if it is present. That does not mean there is an intrinsic ethical difference between the preparations.

13. Some might hold the opposite view—that it is more urgent or important to stop treatment that has been started, since the child has already been harmed by it.

14. In situations like Case 2, Theo’s parents expressed a strong desire for treatment both before it was started and after it was provided. It would be wrong to assume that their desire would be stronger once it had been provided, or to assume that Theo’s parents’ desire for the treatment is stronger than the desires of other families who are want this treatment for their child (but may not have access to it).

15. There is some evidence that views about equivalence may be tractable to teaching or experience. A survey of faculty members with an interest in ethics at the World Critical Care Congress found that the majority (16/22) endorsed equivalence (Sprung et al. 2014). In a survey of U.S. health professionals, those with a high level of experience of end-of-life decisions were 50% more likely to endorse equivalence (Chung et al. 2016). There is a separate need to consider whether or how withdrawal aversion in patients and families should be addressed.

16. This suggestion is similar to the suggestion of Ursin that withdrawal be reframed as “postponed withholding.” We do not believe that it is necessary to frame WD in this way; however, it may be helpful for families and professionals, and may make WD psychologically easier.
